# The reverse zoonotic potential of SARS-CoV-2

**DOI:** 10.1016/j.heliyon.2024.e33040

**Published:** 2024-06-13

**Authors:** Krista M. Milich, Stephen S. Morse

**Affiliations:** a Department of Anthropology, Washington University in St. Louis, 1 Brookings Dr., St. Louis, MO, 63130, United States; b Department of Epidemiology, Columbia University Mailman School of Public Health, 722 West 168th St., NY, NY, 10032, United States

## Abstract

There has been considerable emphasis recently on the zoonotic origins of emerging infectious diseases in humans, including the SARS-CoV-2 pandemic; however, reverse zoonoses (infections transmitted from humans to other animals) have received less attention despite their potential importance. The effects can be devastating for the infected species and can also result in transmission of the pathogen back to human populations or other animals either in the original form or as a variant. Humans have transmitted SARS-CoV-2 to other animals, and the virus is able to circulate and evolve in those species. As global travel resumes, the potential of SARS-CoV-2 as a reverse zoonosis threatens humans and endangered species. Nonhuman primates are of particular concern given their susceptibility to human respiratory infections. Enforcing safety measures for all people working in and visiting wildlife areas, especially those with nonhuman primates, and increasing access to safety measures for people living near protected areas that are home to nonhuman primates will help mitigate reverse zoonotic transmission.

## 1 Introduction

The mass global transmission of severe acute respiratory syndrome coronavirus 2 (SARS-CoV-2) among humans creates concerns for the potential transmission of the virus to other species and the possibility of additional genetic changes occurring in those species. Studies have already shown the wide range of other animal species that are likely susceptible to SARS-CoV-2 [1], and transmission has occurred from humans to other animals in a variety of mammalian species, including mink, gorillas, lions, tigers, white-tailed deer, hamsters, dogs, and cats [[2], [3], [4], [5], [6], [7], [8], [9], [10], [11], [12], [13], [14], [15], [16], [17]]. With much of wildlife research interrupted due to the coronavirus disease 2019 (COVID-19) pandemic (e.g. the majority of field research on nonhuman primates was paused, postponed, or cancelled in response to COVID-19 [[18], [19]]), it is still unclear the extent to which wild animal populations have become infected with SARS-CoV-2 or will become infected as research and tourism resumes to full capacity. Also uncertain are the impacts infection may have on populations of endangered species and the risks to human health as the virus spreads through more species.

Coronaviruses have long been known for their broad host range and diversity and are classic examples of viruses that have crossed species [20]. This dynamic makes interspecies routes of transmission especially important [21]. As respiratory viruses, they are also a concern to our closest relatives, other primates. Respiratory disease is one of the greatest threats for habituated wild apes [22]. In wild chimpanzee populations, respiratory illnesses have caused significant morbidity and mortality [[22], [23], [24]]. At Gombe, the site made famous by the work of Jane Goodall, clinical signs of respiratory illness were more prevalent than wounding, diarrhea, weight loss, or skin issues [22]. At another site, respiratory illness accounted for 58.6 % of deaths with identified causes [23]. At these and other chimpanzee research sites, respiratory disease outbreaks have been linked to human respiratory infections [23,[25], [26], [27]]. In fact, a human coronavirus has been identified as the cause of a respiratory outbreak in one population of chimpanzees [28]. Similarly, respiratory outbreaks in gorillas have also been found to be of human origin [29] and can have devastating consequences. In a given year, over a third of habituated mountain gorillas are impacted by respiratory disease and these illnesses still account for a significant portion of mountain gorilla death despite medical intervention by wildlife veterinarians [30]. Due to gorilla social networks, once a respiratory pathogen has been transmitted to a group, there is rapid spread within the group [31].

What may be more concerning for human health is when humans transmit pathogens to other species which then become a reservoir for the virus and can pass it back to humans. Yellow Fever Virus (YFV) is an example of such dynamics. YFV is thought to have originated in Africa and was then brought to the Americas via shipping routes, together with its mosquito vector, *Aedes aegypti* [32]. After being brought to the Americas, nonhuman primates became infected with the virus and are now an integral part of the transmission cycle, resulting in devastating outcomes for humans and nonhuman primates alike [32,33]. Unlike nonhuman primates in Africa and Asia, at least some species of monkeys in the Americas become symptomatic from YFV infection; die-offs in wild populations have been linked to the disease [33]. Infected wildlife can also transmit pathogens to previously uninfected populations, such as the introduction of tuberculosis to people in the Americas by seals and sea lions prior to European colonization [34]. With each emerging infectious disease, we must be concerned about transmission to other animals and the potential long-term impacts for human health.

The risks and harms associated with disease transmission to other species are compounded by the possibility that those pathogens may undergo genetic changes in the other species and then pass back into humans in a new form. This pattern is well known with another respiratory disease, influenza. Although the 2009 H1N1 influenza pandemic appeared to have originated from pigs, studies now suggest that humans first infected pigs with a closely related strain of influenza and several introductions from humans to pigs were documented [35,36]. It appears that throughout history, human-to-swine transmission of influenza is probably at least as common as swine-to-human [35]. Transmission has been documented in both directions, thereby closing the circle [37]. Furthermore, the continued reintroduction of H1N1 from humans to swine has led to increased genetic diversity of swine influenza that threatens humans and complicates the creation of flu vaccines each year [35].

In this review, we will discuss previous examples of disease transmission between people and other species and explore the potential risks associated with SARS-CoV-2 infection in wildlife, especially nonhuman primate populations. To adequately address zoonotic disease threats, we must overcome our bias of seeing other species as hosts that threaten people rather than people as hosts that infect other species [35]. We should therefore be concerned about pathogens—in this moment, SARS-CoV-2—passing from humans into other animals, circulating and developing new variants in those populations with consequences for endangered wildlife, and then re-infecting humans.

### 1.1 Context

**Fig. 1:**
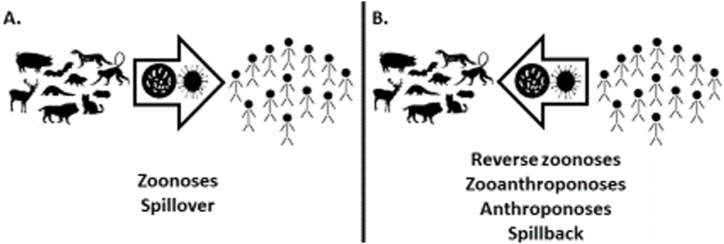
Pathogens that originate in a nonhuman animal source that then infect or “spillover” into humans are referred to as zoonoses (A); whereas, pathogens that “spillback” into other species from a human source are referred to as reverse zoonoses, zooanthroponoses, or anthroponoses (B).

Reverse zoonoses, infections transmitted from humans to other animals, have been documented numerous times before, but have generally been studied less than zoonotic disease transmission to humans [38]. Complicating the issue, terms are used inconsistently in the literature. Reverse zoonoses are sometimes referred to as zooanthroponoses (diseases transmitted from humans to other animals) or anthroponoses (the source of the infection is human), as opposed to zoonoses (the source of the infection is other animals to humans) (Fig. 1) [39,40]. The transmission of diseases between humans and other animals is also referred to as “spillover” (transmission to a human from another species; Fig. 1A) and “spillback” (transmission of a pathogen from humans to another species; Fig. 1B). In this paper, reverse zoonosis will be used to refer to a disease transmitted from humans to other animals.

As with other emerging infections, the consequences of reverse zoonoses can vary [41]. Some may resemble most human “spillover” events, with abortive or self-limited infection. Others may cause small, localized outbreaks of disease with limited transmission. Of greater concern, although likely less common, some may achieve onward transmission in the new host species and establish themselves. “Spillback” to humans, possibly after further evolution in the new host, may also occur. Examples of these are discussed in this paper.

In general, of the human-to-other animal transmission events, there is evidence of multiple routes of transmission, including direct contact, fomite, and aerosols [38]. These will often vary with the pathogen and may resemble the natural routes of transmission. For example, there have been multiple transmissions of tuberculosis from humans to elephants [42] as well as to macaques and other species in contact with infected human handlers [43], the coronavirus found in alpacas is similar to a common human coronavirus (HCoV-229E) which may be due to human-to-alpaca transmission [44], and a study of giardia in humans, livestock, and nonhuman primates in Uganda suggested a human-to-nonhuman primate transmission route [45].

## 2 Synthesis

### 2.1 Reverse zoonotic potential of SARS-CoV-2

**Fig. 2:**
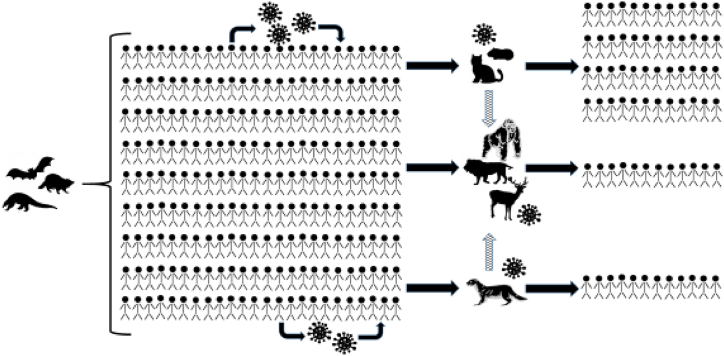
SARS-CoV-2 spillover and spillback events. The outbreak of SARS-CoV-2 in people began after still undetermined zoonotic transmission events, the virus spread quickly around the globe infecting many people. As the virus circulated in human populations, it was also transmitted to a variety of other animals, including pets (e.g. cats and hamsters), wild animals (e.g. deer, lions and other big cats, and zoo-housed gorillas), and farmed animals (e.g. mink). In some cases, SARS-CoV-2 has circulated in populations of these other species and then been transmitted back to humans, including from farmed mink to humans, a zoo-housed lion to a human, and from pet hamsters to humans. New variants of the virus have evolved within human populations and in other species (e.g. farmed mink and wild deer).

Concerns about COVID-19 as a reverse zoonosis are not hypothetical (Fig. 2). SARS-CoV-2 has already infected a number of other species in captivity, including felids, nonhuman primates, and mustelids [46]. At the Bronx Zoo, four tigers and three lions had COVID-19 from at least two transmission events, one of which was from a keeper [2]. Humans were identified as the source of infection in farmed mink, followed by transmission between the mink themselves [3]. The ability of SARS-CoV-2 to infect mink is not surprising. Indeed, the ferret, another mustelid, was the first animal model for influenza [47,48], as well as for SARS-CoV-1 and in some reports SARS-CoV-2 [49]. Based on studies of the main receptor of SARS-CoV-2, the angiotensin-converting enzyme 2 (ACE2), a variety of other animals were found to likely be susceptible to COVID-19, and catarrhine primates (i.e. monkeys in Africa and Asia and apes) had the highest likelihood of susceptibility [1,50]. New variants may be able to infect additional species or result in more severe disease [51]. There is evidence that the Delta variant infected three captive Asiatic lions in India with two of the three having prolonged illness that required treatment with fluids, antibiotics, and nutritional supplements [12]. And the most divergent lineage of SARS-CoV-2 has been identified in wild white-tailed deer in Canada [52].

Cases of COVID-19 in other animals are not always mild. On fur farms, the presence of SARS-CoV-2 was identified based on higher than normal mortality levels [53], and in a zoo setting, a silverback gorilla needed antibody treatment to recover from severe disease [4,51]. Three snow leopards at a Nebraska zoo and a tiger at the Columbus Zoo died of complications associated with COVID-19 [54,55]. Circulation of SARS-CoV-2 in other species also increases opportunities for mutation and for new variants to infect humans. Mink that were infected with SARS-CoV-2 by humans were able to transmit the disease amongst themselves ultimately causing variants to circulate in their own populations [53,56]. There was then transmission of variants from mink back to humans [53,56]. Furthermore, one of the variants (Y453F) was shown to have a much higher affinity for binding than the original SARS-CoV-2 strain, suggesting the possibility of enhanced transmission capacity [57]. Ultimately, the SARS-CoV-2 outbreak in mink included animals on hundreds of farms across multiple continents and even some wild or escaped mink nearby these farms [58]. Concerns that the variants circulating in mink were less able to be neutralized by antibodies in humans led to 17 million mink being killed [59]. These concerns are not unwarranted and could lead to serious ethical dilemmas should a similar type of outbreak occur in an endangered species living in the wild.

Transmission between an endangered species and human has been documented in the case of a zoo-housed African lion [11]. Based on symptoms, repeat testing, and contact records, there is evidence that a lion that was in close contact with zoo staff while symptomatic with COVID-19 transmitted the virus to at least one staff member. Given that the zoo was closed to visitors for the season, the lion most likely contracted the virus initially from a different zoo keeper and then subsequently gave it to other staff who became symptomatic and tested positive three days after close interactions with the sick lion, thus documenting a human-lion-human transmission pattern [11]. Given that all individuals involved (including the lion) were vaccinated and PPE was being used, the findings highlight the cross-species transmission potential when humans interact closely with other species.

Animals that have been infected with SARS-CoV-2 can also cause outbreaks in human populations. A COVID-19 outbreak in Hong Kong was linked to infected hamsters that were imported from the Netherlands and sold as pets, highlighting the risk of transporting the virus internationally in infected animals [5]. These examples show the possibility for animals infected with SARS-CoV-2 from humans to circulate the virus within their population and then re-infect humans either in the same place where the initial reverse zoonosis occurred or in a new location where the animals have been transported. In free-ranging animal populations, this means that the original or new variants could be transmitted back to humans throughout the animal's natural range.

Opportunities for SARS-CoV-2 to be transmitted and evolve in wild populations may be diminished compared to captive animals that are in closer proximity and kept indoors; however, there are still risks. Studies in ferrets found that SARS-CoV-2 could be transmitted over a meter [49], suggesting that even in captive populations of animals maintained at low density, the virus can still be transmitted and have the opportunity to evolve. Although human-animal-human transmission events have been documented, we are still lacking data on the extent to which humans have infected wildlife with SARS-CoV-2 and potential transmission events from these populations back to humans. There is evidence that farmed animals have transmitted the virus to wild-living animals of the same species [58] and that pet animals that roam freely in wildlife habitats are carrying SARS-CoV-2,^60^ making it possible for humans to either infect wildlife with SARS-CoV-2 directly or through their domesticated species.

SARS-CoV-2 has already been found circulating in one species of wild animal—white-tailed deer (*Odocoileus virginianus)* in North America [[13], [14], [15],^17^,^61^]. Of the deer that have been tested, more than one-third have been positive for SARS-CoV-2 antibodies or RNA [[13], [14], [15]]. Multiple strains are circulating in the wild deer population, and there is evidence of over 100 transmission events from humans to deer, followed by deer-to-deer transmission [14,15,17]. During a 5 month period, there were at least 30 transmission events from humans to white-tailed deer in Ohio, USA [10]. Furthermore, during early waves, peaks in positive samples from deer coincided with peaks in human infections and the timing of hunting season [14]. Transmission in deer can occur through direct contact or through vertical transmission from doe to fetus [62]. Variants of concern can persist in deer populations long after their last detection in humans in a given area and highly divergent variants from human strains are also circulating in deer [10,63]. SARS-CoV-2 evolution in white-tailed deer is three-times faster than in humans and is impacted by different selective pressures [10]. Thus, wild populations can serve as a reservoir of variants thought to no longer be a concern to human health and also be a source of novel variants of potential concern. Thus far, there is evidence of at least three deer-to-human transmission events in the USA [17], providing further evidence that human-wildlife-human transmission is a concern.

In India, a wild leopard (*Panthera pardus fusca*) was found to be infected with SARS-CoV-2 during routine postmortem testing [64]. The leopard lived in a protected area that shares a border with human communities, but his case did not coincide with peaks in human infection rates [64]. Even when wild animals do not have direct contact with humans, domesticated animals could spread SARS-CoV-2 infections from human communities to wild populations. For example, in Ecuadorian Amazonia, free roaming dogs tested positive for SARS-CoV-2 leading to concerns about potential transmission to wild animals in the Amazon [60]. The lack of surveillance makes it difficult to know what other wild populations have been infected with SARS-CoV-2 or the extent of transmission among wild populations; however, the existing evidence suggests that these events can occur and will become more frequent with increased human-wildlife interactions. Furthermore, once SARS-CoV-2 has been transmitted to another mammalian species, the virus can easily begin circulating in those populations [65].

There is already substantial evidence that domesticated animals are a concern for becoming infected with SARS-CoV-2, transmitting the virus within their populations potentially leading to mutations, and transmitting existing and new variants to humans [9,[66], [67], [68]]. Although the threat of zoonotic disease transmission of COVID may be greater between domesticated animals and humans, the potential for humans to infect wildlife populations and the potential ramifications of those events have received less attention and should be considered. In fact, the Food and Agriculture Organization, World Organisation for Animal Health, and World Health Organization released a joint statement urging that we prioritize monitoring SARS-CoV-2 infection in wildlife [69]. Through experimental inoculation, a variety of wild animals that overlap in space and time with humans have already been shown to be susceptible to SARS-CoV-2 and able to shed the virus, including Mexican free-tailed bats [70], red foxes [71], raccoon dogs [72], skunks [73], and peridomestic wild rodents [73,74].

Host species vary in a number of ways that can impact the susceptibility of a species to become infected with a particular pathogen and for that pathogen to circulate within a population [75]. The risk of infection for any individual animal within a given wildlife species is linked to both exposure risk and immunogenetics, which are important considerations for mitigating disease spillover [76]. A combination of both host and pathogen characteristics, as well as ecological conditions and host evolutionary relationships can impact pathogen spread and the amplification or dilution of infection prevalence [77,78]. Pathogen infection can also be associated with host behavioral changes that are advantageous to the parasite and facilitate transmission [79]. Host switching can lead to diversification of pathogens [78,80]. RNA viruses, in particular, are known culprits of emerging human diseases that can pass between species [81]. These multi-host viruses can evolve differently in each host [82]. In any given environment, hosts species can carry genetically differentiated variants of a given pathogen, but those pathogens are able to be transferred between hosts provided that the host possesses the necessary target receptor – in other words, pathogens are moving between receptors regardless of species, and taxonomic divisions do not necessarily represent a barrier to a pathogen [81]. Although areas with high COVID-19 rates in human populations have found infection in wildlife populations, the extent of wildlife infection globally is unclear. In certain parts of the world where RT-PCR methods were used to test samples from wildlife, the findings suggest that there are areas where wildlife have not yet been impacted by SARS-CoV-2; however, these studies would benefit from the use of methods that can test for previous infections, rather than single time points [83,84]. As SARS-CoV-2 continues to circulate in human populations during a time when people are resuming travel, wild nonhuman primate populations are of particular concern for reverse zoonotic infection with COVID-19 leading to further zoonotic transmission of the virus.

### 2.2 Potential SARS-CoV-2 threats to nonhuman primates

**Fig. 3:**
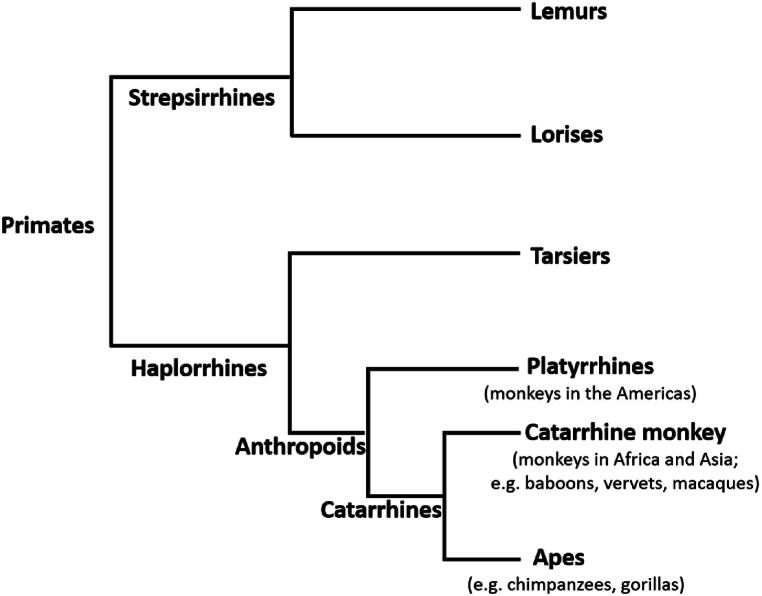
Catarrhine primates include monkey and ape species that evolved in Africa and Asia, including humans.

Among wildlife species, nonhuman primates are of particular concern because they 1) are susceptible to and suffer consequences of respiratory illnesses transmitted from humans [22], 2) can become part of disease transmission cycles that infect humans [32,33], 3) have already been shown to be susceptible to infection and severe disease from SARS-CoV-2 transmitted from humans [51], and 4) have the highest likelihood of susceptibility based on the main SARS-CoV-2 receptor site [1,50]. The close phylogenetic relationship of humans to other primates and the increased rates of human-nonhuman primate interactions have long been a concern for potential pathogen exchange [85]. In a review of human-to-wildlife transmission events of known pathogens, Fagre et al. [86] found that nonhuman primates are more likely to become infected with pathogens from humans and that these pathogens can cause morbidity and mortality in new wildlife hosts. Given the high likelihood of susceptibility of catarrhine primates to SARS-CoV-2 [[1], [50]], these species are of particular concern (Fig. 3). Fischhoff et al. [87] combined data on viral binding with biological and ecological data to predict each species likelihood of becoming a host of SARS-CoV-2. All of the nonhuman primates included in their study were predicted to have high susceptibility as hosts [87]. Many nonhuman primates are also endangered, including all of our closest evolutionary relatives, other apes. Although there currently is not information about COVID-19 in wild nonhuman primates, studies of other respiratory viruses in these species provide insight into the threat of SARS-CoV-2 [[22], [23], [24], [25], [26], [27], [28], [29], [30], [31]]. These previous findings suggest that COVID-19 could have devastating impacts on wild ape populations.

Although less research on respiratory illness has been conducted in nonhuman primate populations in the wild other than apes, there are still many reasons to believe that COVID-19 also poses a serious threat to these species. In biomedical research, various monkey species have been experimentally infected with SARS-CoV-2, and although the suitability of these species as animal models for vaccine development and other treatments is questionable [88], the susceptibility of these species to the virus is evident [[89], [90], [91], [92], [93], [94], [95], [96]]. For example, rhesus macaques, cynomolgus monkeys, baboons, and African green monkeys have been shown to be susceptible to SARS-CoV-2, become symptomatic, have virus replication, and shed virus particles [[89], [90], [91], [92], [93], [94], [95], [96]]. Notably, among the nonhuman primates that have been inoculated in captive settings, a platyrrhine species, the common marmoset, develops milder, if any, symptoms associated with infection [[89], [93]].Although marmosets could develop a fever in response to infection, they did not develop respiratory symptoms [97]. The mild response in marmosets is thought to be associated with differences in the ACE2 receptor [93]. Thus the predictions about which nonhuman primates are of most concern for infection with SARS-CoV-2 based on the ACE2 receptor genetics is consistent with the observed response of infected animals in the lab [1,50,89,93,97].

Transmission of SARS-CoV-2 from humans to wild populations of monkeys could have serious implications for those species, other species they interact with, and human health. There has been relatively little research on the impact of humans on disease risk in wild primates compared to the risk *to* humans from nonhuman primates [98], but as has been seen with other illnesses, nonhuman primates can be both susceptible to and reservoirs of infectious diseases that threaten human populations. In addition to the ACE2 similarities in monkeys living in Africa and Asia that likely make them susceptible to COVID-19 [[1], [50]], many of these species also have high rates of interaction with human populations. For example, Schmitt et al. [99] noted the range of vervet monkeys across Africa, their close proximity to people, and their ability to become infected with SARS-CoV-2 presented a concern for cross-species transmission. Monkeys like vervets, baboons, and macaques that are widespread and are often in close proximity to human communities have the potential to not only be impacted by COVID-19, but to become reservoirs or mixing vessels of the virus. Even forest-dwelling nonhuman primates that are often thought to not overlap significantly with humans do have interactions with people during crop foraging events, hunting activities, and extractive practices [100], which has resulted in the transmission of pathogens between these species and humans [45]. In addition to direct transmission between humans and nonhuman primates, other species could also be intermediaries resulting in transmission of pathogens in either direction. Many wild primates live in areas with other species known to be susceptible to SARS-CoV-2 infection, such as large cats, who could also be part of transmission pathways between humans and other primates.

In a systematic review of reverse zoonoses, Messenger et al. [38] found that viruses were one of the most common pathogens transmitted from humans to other animals. At the same time, primates are more likely to serve as a source for pathogens that can infect humans [86]. Given the close phylogenetic relatedness of humans to other primates, novel pathogens from nonhuman primates may pose a greater risk to public health [41], and these species are also likely susceptible to the viruses circulating in humans. Wild primate populations with a high susceptibility for a novel virus are then an important consideration for trying to take a holistic approach to addressing the ongoing pandemic.

### 2.3 Interventions

The One Health approach, which acknowledges the interconnectedness of human health, the health of other animals, and the environment, is vital to addressing the current SARS-CoV-2 (COVID-19) pandemic and mitigating future pandemic threats [[101], [102], [103], [104]]. The health of humans is inextricably linked to the health of other animals and the environment. Many wild populations were protected from infection by border closures, national park closures, and reduced research and tourism, but these closures have also had negative consequences for conservation [105]. Nevertheless, as travel resumes, the impact on wildlife and disease dynamics must be a primary consideration. One key component of this effort is the messaging and protocols related to ecotourism or wildlife tourism. Tourists often underestimate the risk they pose to wildlife [106]. Tourists engage in a number of behaviors when tracking and observing wildlife that could provide opportunities for disease transmission, including touching their face and then touching forest items such as tree trunks or leaves, eating, coughing, sneezing, and urinating and defecating in the forest [107]. Tourists also do not adhere to distance requirements (e.g. remaining at least 7 m from mountain gorillas) and report that they might still go out in search of endangered species when sick despite strict rules against doing so [108,109]. Importantly, though, these behaviors can at least partially be influenced by the messaging that tourists receive prior to their excursions. One study found that if tourists are told of the devastating effects of not following the rules (such as gorillas getting sick and dying as a result), they are more likely to follow the rules than if they receive a neutral message or a positive message about how compliance prevents disease transmission and death [110].

Wildlife authorities and field sites have implemented some safety rules, such as wearing face masks, hand washing, temperature checks, and distance requirements from wildlife; however, risks still remain despite these safety measures. For COVID-19, in particular, temperature checks are of limited use given the high rate of asymptomatic spread of SARS-CoV-2. Tourist and research sites should also consider mandatory COVID-19 vaccination and booster requirements, quarantine, and SARS-CoV-2 testing for individuals that will be visiting nonhuman primate habitats. These measures, in addition to masking with high quality masks, distancing, and sanitation requirements can greatly reduce the risk of humans transmitting SARS-CoV-2 and other respiratory pathogens to endangered species in these settings. Proper mask wearing, in particular, could be important for tourists and visiting researchers to protect wildlife and local communities [111]. However, humans are not only in close proximity to nonhuman primates during research and tourism, but also through being active in extractive processes, such as mining for oil, gold, silver, and coltan, clearing land for agriculture, and logging forests for timber. These extractive processes are occurring at large scales in the tropics where nonhuman primates live. Ultimately, these operations that temporarily or permanently bring large numbers of people into closer proximity to wildlife present serious concerns for disease transmission both to and from humans. All of the recommended safety measures for researchers and tourists (vaccination, testing, masking, quarantine, and sanitation) should also be enforced for people working in wildlife areas, or even better, these large-scale extractive activities should be significantly reduced.

Aside from these commercial efforts, there are also increased rates of interactions between people and nonhuman primates during individual efforts to extract resources. For example, pole collecting, foraging, and hunting by local community members around a forest in Uganda significantly increased the likelihood that the person would have at least one encounter with a nonhuman primate [112]. Understanding human behavior, including hunting practices, prey choice, and foraging decisions, among other practices that put people in close contact to wildlife, is critical to the development of sustainable interventions to reduce disease emergence [113]. Preventive measures should be taken to reduce susceptibility to zoonotic disease for local community members living near wildlife areas and/or people who rely on wildlife in their diet. There is a need to provide vaccine access to the dense human populations that live near wildlife areas. High host density is associated with more opportunities for pathogens to be transmitted both within a species and to other species [75]. Unfortunately, inequities in vaccine distribution have been a serious concern since the vaccines were developed. Countries like the United States were promoting widespread COVID-19 vaccine boosters with the original vaccine formula while there was still poor access to vaccines globally. In most countries that are home to nonhuman primates, there is less vaccine access than in other countries, and most of the vaccines are being distributed in urban centers rather than in rural areas where people are living in close proximity to wildlife. There is also a lag between updated vaccines for circulating variants becoming available and being accessible to people living in close proximity to wild nonhuman primate populations. Officials in charge of vaccine development and distribution in the United States argue that increased global distribution of COVID-19 vaccines is essential to preventing the evolution of additional variants [114]. It is also worth noting that if novel variants emerge in wildlife, current versions of the vaccine may not be effective against these new variants. Providing quality masks to individuals and communities who cannot easily access them or afford them is also key to reducing transmission.

Additionally, providing One Health education to people living in areas where humans and wildlife overlap is critical to reducing zoonotic transmission events [115]. Providing a reliable source of information that community members can rely on for assessing the threat of COVID-19 and making decisions about safety behaviors can lead to better compliance with safety behaviors, which would reduce the chances of transmission of SARS-CoV-2, as well as other pathogens [116]. The health and well-being of community members living near wild primate populations can also benefit from community-driven projects that reduce the occurrence of crop foraging and prevent wildlife from crossing out of protected areas onto private land [[117], [118], [119]]. Combined, these measures will not only reduce the mutation of variants in human populations, but also the potential for mutations to occur in other species after transmission from humans to other animals.

Safety measures are important, but we must acknowledge that they likely will not be sufficient to completely prevent transmission to wildlife. At Zoo Atlanta, members of the western lowland gorilla group tested positive after showing symptoms of COVID-19, and the zoo released a statement indicating that they believe the gorillas were infected by a COVID-positive staff member who was vaccinated, wearing personal protective equipment, and not symptomatic [120]. One approach to reducing zoonotic transmission of SARS-CoV-2 between humans and other animals is to vaccinate the animals [[121], [122], [123], [124]]. This approach has been taken with a variety of captive animals [123]. However, vaccination will not necessarily prevent transmission to and between other animals—in the case of the human-lion-human transmission event at the zoo, all parties, including the lion, were vaccinated [11]. Furthermore, as with any wildlife vaccination program, this approach is likely to be controversial and logistically difficult. Instead, it may be more prudent to acknowledge that as long as there are high rates of COVID-19 in human populations, there is danger of transmitting the virus to other species in which it can circulate and evolve. Nonhuman primates are of particular concern. In addition to vaccination, sanitation, quarantine, testing, distancing, and masking, we must make efforts to expand disease surveillance in wild nonhuman primate populations and expand healthcare access to people living in closest contact to them. Banerjee et al. [125] argued that because nonhuman primates have the greatest ACE2 similarities to humans, surveillance of potential transmission events should be implemented in areas with high rates of human-nonhuman primate interactions. Targeted surveillance at the human-wildlife interface in areas with high potential for zoonotic disease emergence is key to a comprehensive plan to address the increasing frequency of emerging infectious diseases [126].

Given the likelihood of transmission to wildlife, potential spread and mutation of the virus in wild animals, and subsequent transmission back to humans, there is a need for surveillance and monitoring strategies for SARS-CoV-2 in wildlife [127,128]. Much of the current emerging infectious disease work is passive—identifying new pathogens of concern only after they have infected people. The COVID-19 pandemic highlights the need to take a more strategic approach to monitoring for pathogens of concern, including new variants of SARS-CoV-2 and expanded range of the virus. The One Health approach will allow researchers to identify areas of greatest concern for reverse zoonotic spread of SARS-CoV-2 to wildlife and the potential for sustained transmission and possible mutation of the virus in these populations prior to reinfecting humans. As described here, apes and other nonhuman primates are of high concern for becoming infected, maintaining the illness in their population, suffering devastating consequences for endangered species, and transmitting the virus back to humans.

Under the right circumstances, wild primates could serve as a transmission route for the virus into uninfected communities similar to how hamsters caused an outbreak in Hong Kong [5]. For example, wildlife tourism could result in wildlife becoming infected with SARS-CoV-2 in areas where the local communities have previously avoided outbreaks through lockdowns and border closures. There is the added concern that this type of outbreak would impact communities where healthcare is limited and people have not had access to vaccines. Such an outbreak could further have global ramifications given the likelihood of new variants evolving under these conditions. For these reasons, all possible precautions should be taken to reduce the likelihood of infecting wildlife populations and animals in areas with possible transmission events should be monitored for SARS-CoV-2 variants as part of comprehensive surveillance program.

### 2.4 Outlook

At a larger scale, we must change the destructive and invasive interactions we have with wildlife to protect both ourselves and other species. In recent decades as humans have altered land use patterns and interactions with other animals through intensive habitat modification and mass factory farming, we have seen a corresponding increase in emerging infectious diseases of zoonotic origin [41,129]. Outbreaks of novel viruses are occurring at increasing rates and are resulting in larger outbreaks than in previous years [128]. RNA viruses, such as SARS-CoV-2, are particularly successful at spread between species [126]. Given that zoonotic diseases will likely continue to be a major concern for society and human health, the recommendations we provide here will also be relevant during future outbreaks [101]. This increase in diseases has significant economic and public health repercussions. Increased interactions with wildlife and disease transmission from humans also have devastating effects on wildlife, including endangered species [130]. COVID-19 offers opportunities for societal changes including changing the way we interact with the natural world [131]. Ecosystems provide resilience to current and future zoonotic threats [131]. However, rather than benefiting from the protections provided by ecosystems, we are increasing risks to our own health and safety through our destruction of these ecosystems. As the COVID-19 pandemic has exemplified, these actions pose a threat to society. We should make efforts to minimize contact between humans and other animals when possible and stop the destruction of wild areas. Calculations of the global economic burden posed by zoonotic diseases should be incorporated into the market value of commodities such as palm oil, beef, gold, and oil, among others. Furthermore, we must acknowledge the role of humans in zoonotic disease cycles and remove the bias of seeing humans as the victims of these outbreaks rather than active participants in the process. In that way, it may be time to stop differentiating between a “zoonosis” and a “reverse zoonosis” and instead recognize humans as one of the animals that are contributing to zoonotic disease spread. The transmission of SARS-CoV-2 from humans to other animals could lead to a future pandemic resulting from a new and devastating descendent of the current virus that evolves in one of the other animal hosts that we are currently putting at risk of infection.

The COVID-19 pandemic led to rapid, widespread societal change in response to concerns for public safety, making it clear that these sorts of mass societal changes are possible. However, many of the threats to public health have remained unaddressed, including access to social services for those in need, equity in access to healthcare, and mitigation strategies to prevent climate change and biodiversity loss that are linked to health consequences. Without these measures, people will continue to suffer from both the current and future pandemics.

## Data availability

No data were generated in the writing of this article.

## CRediT authorship contribution statement

**Krista M. Milich:** Writing – review & editing, Writing – original draft, Conceptualization. **Stephen S. Morse:** Writing – review & editing, Writing – original draft, Conceptualization.

## Declaration of competing interest

The authors declare that they have no known competing financial interests or personal relationships that could have appeared to influence the work reported in this paper.
